# Imaging of biofilms using cryo-scanning electron microscopy

**DOI:** 10.1042/EBC20250032

**Published:** 2026-09-02

**Authors:** Saskia E. Bakker

**Affiliations:** Advanced Bioimaging RTP, University of Warwick, Gibbet Hill Road, Coventry CV4 7AL, U.K.

**Keywords:** biofilm, cryo-scanning electron microscopy, electron microscopy, experimental design, workflow

## Abstract

Biofilms are challenging samples for microscopy, because they are usually large samples with small features of interest, a fragile nature due to their high water content, and they may be grown on a variety of substrates. Cryo-scanning electron microscopy (cryo-SEM) has long been used to image microbiological components in plant–pathogen interactions, food products, and soil. The main advantage, compared with the more widely available room temperature SEM, is that the rapid sample preparation results in a native-like structure. It has been used to study environmental biofilms, but rarely for medically relevant biofilms.

The cryo-SEM workflow starts with freezing the sample, either by high-pressure freezing, plunge-freezing, or slush nitrogen freezing. This is followed by fracture, sublimation, and coating, before the cryo-SEM imaging takes place.

The present review aims to give new potential users an overview of the workflow and give examples of the equipment available, while discussing advantages and limitations of specific steps and their suitability for the research of various biofilms.

## Introduction

From an imaging perspective, biofilms are challenging samples. The combination of the sample size, often in the centimetre range, with the features of interest on the order of micrometres or smaller, means that you need a combination of large field of view and high resolution. Mature biofilms can be heterogeneous and fragile, so an imaging method with minimum interference is desirable.

In addition, biofilms under investigation are often grown on either their natural substrate or in a model that closely mimics their natural environment, such as catheters, endotracheal tubes [1], modified metal surfaces, minerals [2], soil [3], and biological substrates [4].

Cryo-scanning electron microscopy (Cryo-SEM), also referred to as low-temperature SEM, is a technique commonly used to image plant material [5], food products [6,7], and hydrogels [8,9]. It has also long been used to study microorganisms in soil [10].

Room-temperature, or ‘conventional’, scanning electron microscopy (SEM) is suitable for imaging bulk samples but takes place in a vacuum that requires the samples to be dehydrated. Hydrated samples would boil in the vacuum, destroying the sample and contaminating the microscope [11]. This presents another challenge for biofilm imaging, since biofilms rely on water for a lot of their structure and drying the sample often causes extensive flattening. While room temperature SEM can give useful information about biofilm structure, the sample preparation and dehydration steps can remove loosely associated features and alter the structure. A comprehensive account of sample preparation procedures for room temperature SEM can be found in Bozzola [11] or Arunachalam and Davoodbasha [12]. Modifications to the routine protocols with additional fixation steps, including alcian blue, ruthenium red, and tannic acid, can help with improved preservation of the extracellular polymeric substance (EPS) and biofilm matrix, which allowed for examination of inter-strain variations in matrix, although further optimisation may be required for different bacterial species [13].

For *Pseudomonas aeruginosa*, when imaged under room temperature conditions the bacteria had different proportions to those imaged under cryo-conditions, with non-isometric shrinkage along the long and short dimension of the cell, resulting in a different cell shape [14]. Similarly, Dohnalkova [15] showed severe shrinkage of bacterial EPS in samples prepared for room temperature imaging of *Shewanella oneidensis* compared with cryo-SEM and cryo-TEM. While shrinkage of the cell itself was also observed, this was more isometric than in *P. aeruginosa*, suggesting that different species may react differently to dehydration, making it challenging to extrapolate from room temperature data.

### Cryo-SEM in practice

**Figure 1:**
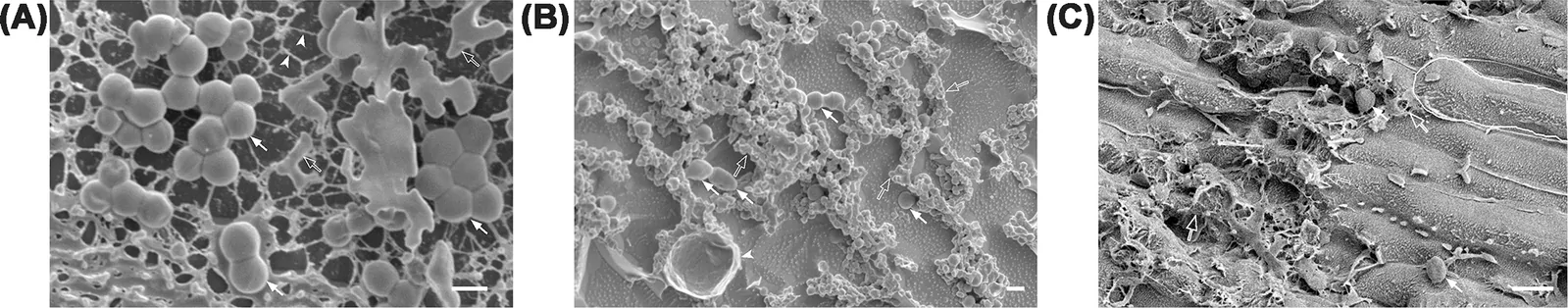
Examples of cryo-SEM images (**A**) Biofilm of *Staphylococcus aureus*, showing clusters of bacteria (indicated with solid white arrows) on a background of EPS (outline arrows) and hydrogel matrix (arrowheads). Scale bar 1 μm. (**B**) Cryo-SEM image of yoghurt, showing microorganisms (solid white arrows) in a protein aggregate. The notched arrow indicates a milk fat globule. Scale bar 1 μm. (**C**) Environmental biofilm showing long cyanobacterial chains (example indicated in white outline on the right) coated in EPS (outlined arrows), as well as single microorganisms (solid white arrows). Scale bar 5 μm.

Figure 1 shows some examples of biofilm images obtained by cryo-SEM. As a logical progression from its long-standing use in imaging plant material, cryo-SEM has been used to study various interactions of plants and microorganisms, often showing biofilm-like properties. When investigating spinach leaves with naturally occurring bacterial infections, cryo-SEM was used to image the bacterial colonies, including detailed studies of the interaction of the EPS with the leaf surface. Additionally, bacteria were able to grow from the frozen samples [4]. Fungi and bacteria in the soil associated with wheat roots were studied and the mucilage (EPS) in this environment could be seen [10]. More recently, surface biofilms from tidal estuary mud were shown to contain EPS and clay–EPS complexes, highlighting the importance of a biologically relevant environment when studying biofilms [3]. Cryo-SEM was used to show a matrix of EPS for aggregating strains of the plant pathogen *Xanthomonas campestris*, which was absent from planktonic strains [16]. In the fungus *Knufia petricola*, which forms biofilms on rock surfaces and plays a role in rock weathering, there was more EPS visible in melanin-deficient mutants than in the wild-type strains [2]. In an endotracheal tube model, cryo-SEM showed that biofilms of *Pseudonomas aeruginosa*, *Klebsiella pneumoniae*, and *Candida albicans* grown in synthetic ventilated airway mucus have a different morphology compared with those in standard medium. This provides a more realistic system for testing the susceptibility of these biofilms to potential treatments [1]. During development of potential diagnostics for biofilm infections, cryo-SEM was used to show degradation of specifically formulated hydrogels by bacterial activity, which confirmed the spectroscopic data [17].

### How does it work?

**Figure 2:**
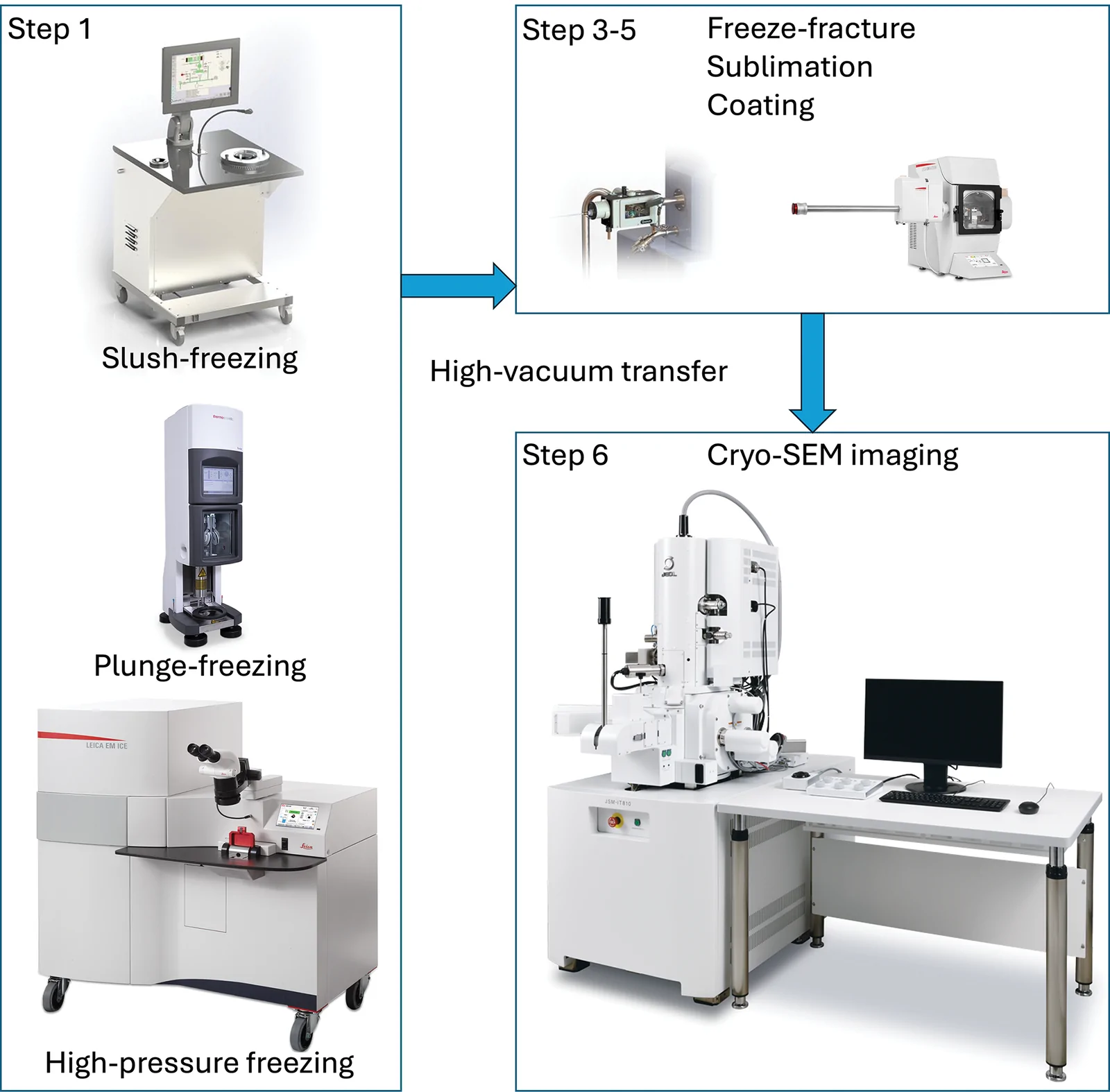
The cryo-SEM workflow This figure shows examples of the equipment that is used for each step. Equipment may vary depending on the manufacturer and model. Equipment shown is (Step 1) slush-freezing: Quorum Technologies Prep-dek^®^; plunge-freezing: ThermoFisher Vitrobot; and high-pressure freezing (HPF): Leica Microsystems EM ICE. (Steps 3–5) left: Quorum Technologies PP3010 Cryo Preparation Chamber; right: Leica Microsystems EM ACE600 with EM VCT500. (Step 6) JEOL JSM-IT810. Images reproduced with permission from JEOL U.K., Leica Microsystems GmbH, Quorum Technologies, and ThermoFisherScientific.

The cryo-SEM workflow consists of six steps: sample freezing, sample transfer, an optional fracture or polishing step, sublimation, metal coating, and finally imaging. Depending on the manufacturer of the equipment, some of these steps may be combined in a single instrument. The workflow and examples of the equipment are shown in Figure 2.

### Step 1: freezing of the sample

The goal of sample preparation for cryo-SEM is to preserve the structure of the sample in as natural a state as possible. This means that we want to avoid artefacts from chemical sources, such as fixatives and cryo-protectants, as well as damage caused by ice crystals. When ice crystals form, the water expands, which causes damage to surrounding biological structures and changes their appearance.

The ideal situation is freezing the water in the sample so fast that there is no time for ice crystals to form. In this case, the frozen sample is amorphous or ‘vitreous’. At ambient pressure, true vitrification only happens at cooling rates in the order of −10^5^ K/s. Due to restrictions in heat transfer within the sample, these rates can only be achieved for a thin sample or the thin outer layer of a larger sample [18,19]. An acceptable and practical compromise may be the formation of nanocrystalline ice, where the size of the ice crystals does not visibly disrupt the structure of the sample at the resolution used for imaging [5]. Crucially, submerging the sample in liquid nitrogen does not provide a high enough cooling rate to prevent ice crystal formation due to the Leidenfrost effect [20]. Although a variety of freezing devices and several cryogens have been tried in the past, only three well-established methods are now commonly used.

#### Plunge-freezing

During plunge-freezing, the sample is held in tweezers and rapidly plunged into a liquid cryogen, usually ethane or a mix of ethane and propane. The method was pioneered by Jacques Dubochet, who won a share in the 2017 Nobel Prize for Chemistry for that work [21]. The devices are designed around pipetting an aqueous sample onto a TEM grid held in tweezers and blotting off excess liquid to leave a very thin layer of liquid sample, which is then plunged into the cryogen. In its simplest form, the equipment is a weighted rod with a release mechanism to drop the tweezers into the cryogen below. Thanks to developments within the cryo-TEM community, several semi-automated commercial devices are on the market, which usually include a temperature- and humidity-controlled chamber for the samples, automated blotting for excess liquid, and a timing system for the process.

Plunge-freezing has been shown to be capable of producing vitreous ice throughout a mammalian cell monolayer when this is grown on TEM grids [22]. Mammalian cells have also been successfully plunge-frozen in ethane/propane when grown on glass coverslips, and these were then imaged by fluorescence expansion microscopy [23]. Although cells on coverslips may not actually be vitrified using plunge-freezing, this method provides a useful example of carriers other than TEM grids being used. This shows that the method is adaptable to biofilm research, especially if the samples of interest can be grown on coverslips or similar substrates.

Although plunge-freezers are widely available within the cryo-EM community, the primary limitation for biofilm applications is whether samples will fit into the cryogen container and through any ports that separate the chamber from the cryogen. Additionally, larger substrates or substrates with a larger heat capacity may cause the cryogen to evaporate.

#### Slush nitrogen freezing

Slush nitrogen freezing is commonly used as the preparation step for cryo-SEM of plant material [5]. Although liquid nitrogen does not provide rapid enough cooling due to the Leidenfrost effect, it is possible to reduce the nitrogen boiling and improve the freezing rate by applying a vacuum to liquid nitrogen. This forcibly extracts energy and causes the nitrogen to solidify. Releasing the vacuum results in a mixture of solid and liquid nitrogen that allows for rapid cooling of a sample [19]. Although the depth of true vitrification is limited, ice crystals usually remain small enough that this method is suitable for certain applications [5], such as freeze-fracture (see Step 3). Although this method can in theory be carried out with a single vacuum pump and bell jar [19], two dedicated commercial devices are on the market as part of a cryo-SEM workflow. The sample carriers are flexible and can be adapted to various sample types. Sample size and thickness are still a consideration and some substrates may pose a problem if they provide thermal or electrical insulation from the carrier and stage. Faster freezing rates can be obtained by using a smaller sample holder such as a rivet instead of the cryo-shuttle [24].

#### High-pressure freezing

For HPF, the sample is mounted between two metal discs with a small recess. This ‘sandwich’ is then further enclosed in a plastic cylinder. A pressure of 1900–2100 bar is applied to the sample followed by freezing with jets of liquid nitrogen. The high pressure reduces the freezing point of water, slows ice crystal nucleation, and prevents the expansion required to turn liquid water into crystalline ice [18,20,25]. The process requires careful synchronisation of the steps and the equipment is necessarily rather complicated and involves a large volume of liquid nitrogen.

HPF can produce well-frozen samples up to 200 μm thick and is widely accepted to provide the best preservation of cellular ultrastructure structure [26,27]. However, the sample size is generally limited to 6 mm in diameter [17], which makes it challenging to apply this method to many biofilm samples.

HPF equipment is reasonably common in EM labs, since it is used before freeze-substitution for room temperature TEM and cryo-sectioning for cryo-TEM. Comprehensive reviews of HPF are given by Kaech and Ziegler [26] and Resch [18].

### Step 2: Transfer

After freezing, samples can be stored in suitable containers in a liquid nitrogen storage dewar, especially if it was plunge-frozen or high-pressure frozen, rather than slush-frozen directly on a carrier. At this point, samples can also be transported in a dry shipper, which is commonly used for transporting cryo-TEM samples and frozen protein crystals.

For successful imaging, the transfer of the sample between the freezing apparatus and the freeze-fracture/sublimation/coating chamber needs to maintain the sample temperature below the point where water can crystallise. Additionally, it needs to protect the sample from contaminations, such as surface ice crystals that will form on condensation of moisture from the atmosphere [28]. The cryo-workflow will include a transfer system that can be evacuated to provide a vacuum-to-vacuum transfer. Any manipulation of the sample after freezing, such as release of planchettes from the carrier of the high-pressure freezer, will need to take place under liquid nitrogen [18,24]. Depending on the setup, a second transfer step may be required between coating (Step 5) and imaging (Step 6), but the same considerations would apply.

### Step 3 (optional): freeze-fracture or cryo-planing

Freeze-fracturing the sample before imaging can be used to investigate internal structures. To fracture, the frozen sample is hit with a cooled knife. The sample will fracture along the lines of natural weaknesses within it, which can result in a sample surface with a lot of topographical features, which is well suited to secondary electron imaging (see Step 6) [5]. Although a slush-frozen sample will almost certainly not be vitrified all the way through, a sample with nanocrystalline ice can still give good results in a freeze-fracture experiment [5,24].

For a smoother imaging surface, it is possible to perform cryo-planing on the sample prior to imaging. This is done with a diamond knife in a cryo-ultramicrotome, which allows for better targeting than the more random process of freeze-fracture. Cryo-planing requires a separate instrument and is sensitive to surface contamination during the planing and transfer steps [18,29].

### Step 4: sublimation

A sublimation step removes surface ice, either from adherent water from the specimen or contamination introduced during transfer. Technically, sublimation is a form of freeze-drying and it may also be referred to as etching or freeze etching. The sublimation step enhances membrane definition in fractured or cryo-planed samples, and can also help to provide information about extracellular spaces by improving the differentiation between gas filled and fluid filled spaces [19,30].

The main sublimation parameters are time, temperature, and pressure. A longer time, higher temperature, and lower pressure will each lead to more sublimation. Although more sublimation can give better definition, over-drying during the sublimation step can cause collapse of microstructure and aggregation of proteins. Water sublimation starts when the sample reaches −110°C, so the time taken to reach a higher target temperature such as −90°C and cool down again afterwards adds to the set sublimation time [29].

### Step 5: sputter coating

During SEM imaging, an electron beam is moved along the surface of the sample and resulting secondary or backscattered electrons are detected. In a sample that is not electrically conductive, such as a frozen biological specimen, the electrons build up a charge within the sample, which results in distortions in the image. Additionally, the accumulation of energy from the electron beam may cause a frozen sample to melt and distort. To prevent this, an ultrathin conductive coating is deposited onto the sample. Depending on the equipment available, this may be a metal such as gold or platinum, or a thin layer of amorphous carbon. If subsequent elemental analysis is planned, carbon coating causes less interference than metal coating [24,31].

### Step 6: cold stage and imaging

Finally, the sample is transferred to the cold stage within the SEM chamber for imaging. During imaging, the sample needs to stay cold and disperse the electrons deposited by the electron beam. Therefore, good thermal and electrical conductivity between the SEM stage and the sample are required. The stage also needs to be mechanically stable and free from temperature fluctuations to prevent sample motion during imaging [30].

SEMs are usually equipped with multiple detectors that can be used for imaging the sample, which will each have an optimal working distance and acceleration voltage. The appearance of the image depends on whether the detector detects high-energy backscattered electrons or low-energy secondary electrons, and the physical location of the detector in relation to the sample [31]. Secondary electron detectors are often used to show topographical features of a cryo-sample. From a user point of view, it is most important to keep microscope and detector settings the same between different samples and sessions. The settings are commonly recorded in the image.

### Limitations of cryo-SEM

Despite its many advantages, cryo-SEM still has limitations. It cannot be used on live samples and imaging different time points will require separate samples for each time point. In many electron microscopy facilities, samples presenting a biological hazard will still require some form of inactivation, which may change biofilm structure or add processing steps. Freezing alone is insufficient to reduce the biohazard, since bacteria can be cultured after imaging with cryo-SEM [4], and Erk et al. [32] report that 70% of high-pressure frozen yeast cells remain viable after thawing.

Preparation methods still come with artefacts and the same biofilm prepared in different ways can give different results. The formation of ice crystals also leads to a separating effect where molecules dissolved or suspended in the water are excluded from the forming ice crystal and aggregate. This gives a distorted view of the actual distribution of the suspended molecules [19].

A comparison of air-dried, liquid nitrogen frozen, liquid ethane frozen, and high-pressure frozen samples of *Staphylococcus aureus* biofilms found that the biofilm could rearrange because of crystallisation effects [33]. Additionally, slush-frozen protein hydrogels show a different pore size in the bulk of the sample compared with the edge, indicating formation of larger ice crystals in the interior of the sample [9]. A similar effect may apply in biofilms, especially those with higher water content.

While HPF may give a more native-like structure than freezing in slush nitrogen, as the increased pressure does not allow for the formation of large ice crystals [25,33], it is limited to very small samples. This makes it unsuitable for many experiments investigating biofilms grown on medically or environmentally relevant surfaces.

Although anti-freeze agents, such as saccharose in cryo-sectioning, can be used to improve the depth of effective freezing, they require chemical pre-fixation to improve cell preservation during the penetration of the cryo-protectant, which comes with its own artefacts [20]. However, denser biofilms with naturally high concentrations of polysaccharides and mucins may derive some cryo-protectant effect from these compounds.

### Complementary techniques

Depending on the setup of the microscope, two additional techniques may be available on a cryo-SEM system.

Energy-dispersive spectroscopy (EDS) can be valuable to determine the presence and distribution of elements within a sample, especially when applied to heavier elements not usually present in biological specimens, such as metals. Recent advances in technology make EDS possible on cryo-samples. Generally, the detection limit is in the range of 1% w/w. Sublimation and coating steps may need to be adjusted for better results. A review is provided in Warley and Skepper [34].

Focussed ion beam (FIB) milling can be used to remove material from a sample and investigate further internal structure through cross-sections or site-specific sectioning. Unlike freeze-fracture, FIB milling provides fine control over the area to be removed, although it is limited to small volumes. The poor conductivity of ice means that the sample is susceptible to charging effects. Detailed information can be found in Hayles and De Winter [28].

### Designing your experiment

While the cryo-SEM workflow is flexible to some degree, the equipment that is available in the microscopy facility may determine some of the experimental design. It is worth establishing the options early in the experimental design phase. It may be possible to access specific equipment in other institutions if that is likely to improve the outcome of the experiment.

If the microscope is in a different location than the lab, safe transport of the samples will be required, or samples may have to be cultivated nearer to the microscopy facility.

An important restriction for medically relevant biofilms will be the biological containment level of the microscopy lab, which determines whether chemical or other fixation methods will be required prior to freezing. If transport to a different facility is required, it may be preferable to do this after freezing to preserve the structure of delicate samples.

Choosing a suitable freezing method will depend on the size of the sample and the substrate it is grown on, but also what is available in the microscopy facility. Ideally, the biofilm would be left on the substrate during imaging but at least during the freezing process to avoid mechanical disruption. For each freezing method, a variety of sample holders is available. Examples of holders are given in Morrison [24] for the slush-freezing and Resch [18] for HPF. If freeze-fracture is a desirable option, this also affects the choice of holder.

The running time of the instrument may also be a factor, especially determining the number of samples that can be imaged in a session. Some holders may hold multiple samples, which improves throughput and reduces the cost for a given number of specimens. Even so, imaging large numbers of biological or technical replicates by cryo-SEM may be unattainable or unaffordable.

During a cryo-SEM experiment, the conditions and parameters should be kept the same between different samples. Sublimation and coating parameters may affect the structure of a biofilm, meaning that consistency in these parameters is very important for comparing samples. While not directly affecting the structure, a change in detectors may change the appearance of the sample; therefore, it is important to use the same detector throughout. As the samples are destroyed after imaging, ensure that the recorded images are comparable in magnification and quality before changing the sample.

## Conclusion

In conclusion, while cryo-SEM is a powerful technique for the imaging of biofilms, it is not without its limitations. When designing and optimising the experiment, it is important to keep restrictions, such as sample size and substrate type, in mind from the very start. It is also important to be realistic about what kind of information will be obtained from cryo-SEM and what techniques and equipment are available, either locally or elsewhere.

## Summary

- Cryo-SEM is a powerful and versatile technique that has some unique advantages when it comes to imaging biofilms.
- Cryo-SEM sample preparation retains biofilm structure better and is faster than resin embedding methods.
- Although commonly used for plant pathogens and environmental biofilms, cryo-SEM is under-utilised for medical microbiology.
- When designing experiments, it is useful to take into account the requirements and limitations of any imaging components, such as sample size and substrates.

## Data Availability

Images shown are available on request.

## Open Access

Open access for this article was enabled by the participation of the University of Warwick in an all-inclusive Read & Publish agreement with Portland Press and the Biochemical Society under an agreement with JISC.

## Abbreviations

cryo-SEM: cryo-scanning electron microscopy
EDS: energy-dispersive spectroscopy
EPS: extracellular polymeric substance
FIB: focussed ion beam
HPF: high-pressure freezing
SEM: scanning electron microscopy
TEM: transmission electron microscopy
